# The advances in acetylation modification in senescence and aging-related diseases

**DOI:** 10.3389/fphys.2025.1553646

**Published:** 2025-05-12

**Authors:** Maiqi Xu, Wenbin Wang, Saien Lu, Mengyao Xiong, Tong Zhao, Yao Yu, Chunyu Song, Jinjing Yang, Naijin Zhang, Liu Cao, Guozhe Sun, Sichong Chen, Pengbo Wang

**Affiliations:** ^1^ Department of Cardiology, The First Hospital of China Medical University, Shenyang, Liaoning Province, China; ^2^ Institute of Health Sciences, China Medical University, Shenyang, Liaoning Province, China; ^3^ Department of Pharmaceutical Toxicology, School of Pharmacy, China Medical University, Shenyang, China

**Keywords:** acetylation modification, aging, DNA stability, mitochondrial function, network mechanism, protein homeostasis

## Abstract

Aging is a process in which organisms or cells undergo a decline in their functions. Epigenetic modification changes have been recognized as a senescence hallmark in both natural aging and stimulation-induced senescence. An acetylation modification is a dynamic process, which plays a crucial role in the senescence process through DNA stability, metabolism, and signaling pathways. We summarized the role and regulatory pathways of acetylation modifications in senescence. Various cell fate-determining proteins regulate multiple cellular processes through acetylation modifications. These processes interact and coordinate with each other, forming an integrated regulatory network framework that collectively drives cellular senescence *via* multiple systemic mechanisms. Based on these findings, we proposed the “acetylation-network regulation-cellular senescence” model, to elaborate how acetylation contributes to senescence. We believe this insight could provide new directions and intervention strategies for senescence and aging-related diseases.

## 1 Introduction

Aging is a complex process involving the decline of biological and metabolic activity, leading to the occurrence of various aging-related diseases, such as neurodegenerative diseases, cardiovascular diseases, metabolic diseases, immune system diseases, and cancer (Guo et al., 2023a). Over the past decade, our research has focused on age-related impairments in the cardiovascular system. We have observed that exogenous damaging stimuli frequently induces varying degrees of post-transcriptional modifications (PTMs) in functional and damage-associated molecules. These molecular alterations ultimately determine cellular fate and drive pathological changes in cardiac function. Our investigations have revealed that lactylation modification of α-myosin heavy chains (α-MHC) played a crucial role in maintaining myocardial fiber contractility. In angiotensin II (Ang II)-induced injury models, we observed significant reduction in α-MHC lactylation levels leading to heart failure pathogenesis (Zhang N. et al., 2023). Notably, acetylation modification of Septin4, a key apoptotic regulator, enhanced its pro-apoptotic capacity and exacerbated hypertensive nephropathy, whereas Sirt2-mediated deacetylation effectively ameliorated this pathological progression (Zhang et al., 2023b). Furthermore, during myocardial remodeling processes, ubiquitination-mediated degradation of poly (ADP-ribose) polymerase 1 (PARP-1) was found to attenuate apoptotic damage, thereby alleviating pathological cardiac hypertrophy and heart failure development (Zhang et al., 2020). These collective findings demonstrate that PTMs of critical cell fate regulators are actively involved in cellular decision-making processes under pathological stress conditions, ultimately determining tissue-specific pathological outcomes.

Cellular senescence, a state of irreversible cell cycle arrest, is a hallmark of aging and a key driver of age-related pathologies. Senescent cells accumulate with age due to persistent DNA damage, oxidative stress, or telomere shortening. Senescent cells contribute to the development of age-related diseases through the senescence-associated secretory phenotype (SASP) (Zhang L. et al., 2022). The SASP creates a toxic microenvironment that disrupts tissue homeostasis through multiple mechanisms. For instance, SASP factors like interleukin-6 (IL-6), matrix metalloproteinases (MMPs), and transforming growth factor-beta (TGF-β) promote chronic inflammation, extracellular matrix degradation, and fibroblast activation, contributing to conditions such as osteoarthritis, pulmonary fibrosis, and atherosclerosis (Li X. et al., 2023; Victorelli et al., 2023; Kandhaya-Pillai et al., 2022; Li et al., 2024a). And the acetylation plays critical roles in SASP-mediated senescence process (Li et al., 2024a). For example, the acetate-dependent acetyl-CoA synthetase 2 (ACSS2) drives the SASP and promotes cellular senescence by modulating the acetylation of PAICS (a key enzyme in purine biosynthesis) (Yang et al., 2025). The work by Giorgio et al. also revealed that the competitive interplay between acetyltransferases and deacetylases modulates the acetylation levels of histone (H3K27ac), thereby influencing cellular senescence processes (Di Giorgio et al., 2021). The loss of epigenetic information is recognized as a hallmark of cellular senescence and various age-related diseases, and thus we focus on the role and potential mechanism of acetylation modifications in senescence (Yang et al., 2023).

**FIGURE 1:**
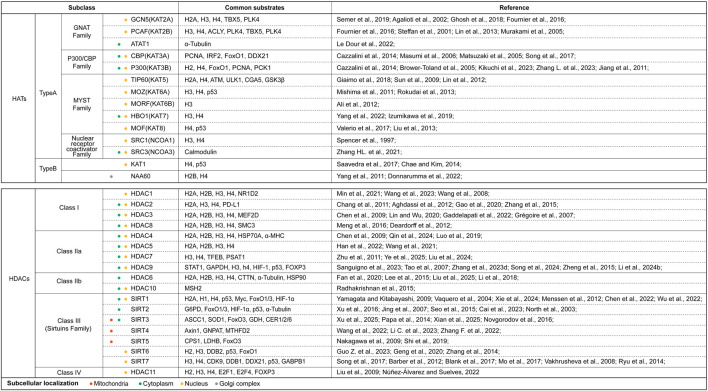
The summary of acetyltransferase and deacetylases. This figure systematically categorized and clustered various HATs and HDACs based on their structural families, subclasses. We also listed their subcellular localization (use different colors to represent different positioning) and main substrates which were reported in current studies. Abbreviations: ACLY, ATP-citrate lyase; ACSS1, acetyl coenzyme A synthetase 1; ATAT1, alpha-tubulin acetyltransferase 1; ATM, ataxia-telangiectasia mutated kinase; α-MHC, α-Myosin Heavy Chain; Axin1, Axis Inhibition Protein 1; CDK9, Cyclin-dependent kinase 9; CER1/2/6, eceriferum 1/2/6; CPS1, carbamoyl phosphate synthetase 1; CTTN, cortactin; CYCS, cytochrome c; DDB1/2, DNA damage-binding protein 1/2; DDX21, DEAD-Box RNA Helicase 21; E2F1/4, E2F Transcription factor 1/4; FoxO1/3, Forkhead box-O-1/3; FOXP3, Forkhead box P3; G6PD, glucose-6-phosphate dehydrogenase; GABPB1, GA-binding protein subunit beta-1; GCN5, general control non-depressible 5; GDH, glutamate dehydrogenase; GNAT, Gcn5-related acetyltransferase family; GNPAT, glyceronephosphate O-Acyltransferase; GSK3β, glycogen synthase kinase 3β; H, histone; HAT, histone acetyltransferase; HDAC, histone deacetylase; HIF-1α/2α, hypoxia inducible factor-1α/2α; HSP70A, heat shock protein 70A; IRF2, interferon regulatory factor 2; KAT, lysine acetyltransferase; LDHB, lactate dehydrogenase B; MEF2D, myocyte enhancer factor 2D; MORF, MOZ-related factor; MOZ, monocytic leukemic zinc finger; MSH2, MutS homolog 2; MTHFD2, methylenetetrahydrofolate dehydrogenase 2; NAA60, N-alpha-acetyltransferase 60; NCOA1/3, nuclear-receptor coactivator 1/3; NF-κB, nuclear factor-kappaB; NR1D2, nuclear receptor subfamily 1 group D member 2; P300/CBP, P300/CREB-binding protein; PCAF, P300/CBP-associated factor; PCNA, proliferating cell nuclear antigen; PCK1, cytosolic phosphoenolpyruvate carboxykinase 1; PLK4, polo-like kinase 4; PSAT1, phosphoserine aminotransferase 1; SIRT, Sirtuins family; SMC, structural maintenance of chromosomes; SOD1, superoxide dismutase 1; SRC1/3, steroid receptor coactivator 1/3; TBX5, T-box transcription factor 5; TFEB, transcription factor EB; TIP60, Tat-interacting protein 60; ULK1, UNC-52-like kinase 1.

Increasing evidence confirms that PTM alterations are widespread in aging and related diseases. PTMs could bring unique changes that can lead to the generation of new phenotypes beyond those encoded by DNA sequences (Sen et al., 2016; Schroeder et al., 2013; Rando and Chang, 2012). Acetylation modification is fundamentally characterized by the covalent conjugation of acetyl groups (O=C-CH3), derived from acetyl-CoA as the biochemical donor, to either ε-amino groups of lysine residues or N-terminal α-amino groups, throughout the peptide chain. Mechanistically, this process is dynamically regulated by the opposing enzymatic activities of histone acetyltransferases and deacetylases (HATs/HDACs), which collectively maintain acetylation homeostasis through precise substrate recognition and catalytic modulation. Emerging research has fundamentally reshaped our understanding of acetylation enzymes. Contemporary studies have revealed that HATs/HDACs exhibit extensive substrate promiscuity, targeting not only histones but also non-histone cytoplasmic and nuclear proteins. Notably, these enzymes demonstrate specific affinity for pivotal cell fate regulators including p53, PARP-1, and signal transducer and activator of transcription 3 (STAT3) (Xia et al., 2022; Dai and Gu, 2010; Zhang N. et al., 2021). This paradigm shift has driven systematic reclassification of acetylation-related enzymes, with current nomenclature emphasizing their catalytic mechanism rather than substrate specificity. Given the exclusive modification of lysine residues, these enzymes are now more precisely designated as lysine acetyltransferases (KATs) and lysine deacetylases (KDACs), reflecting their biochemical specificity and functional diversity. Figure 1 summarized and clustered various enzymes which involved in the acetylation and deacetylation process (Semer et al., 2019; Agalioti et al., 2002; Ghosh et al., 2018; Fournier et al., 2016; Steffan et al., 2001; Lin et al., 2013; Murakami et al., 2005; Le Dour et al., 2022; Cazzalini et al., 2014; Masumi et al., 2006; Matsuzaki et al., 2005; Song et al., 2017; Brower-Toland et al., 2005; Kikuchi et al., 2023; Zhang L. et al., 2023; Jiang et al., 2011; Giaimo et al., 2018; Sun et al., 2009; Lin et al., 2012; Mishima et al., 2011; Rokudai et al., 2013; Ali et al., 2012; Yang et al., 2022; Izumikawa et al., 2019; Valerio et al., 2017; Liu et al., 2013; Kumar et al., 2012; Yang et al., 2011; Spencer et al., 1997; Zhang HL. et al., 2021; Saavedra et al., 2017; Chae and Kim, 2014; Donnarumma et al., 2022; Min et al., 2021; Wang et al., 2023; Wang et al., 2008; Chang et al., 2011; Aghdassi et al., 2012; Gao et al., 2020; Zhang et al., 2015; Chen et al., 2009; Lin and Wu, 2020; Gaddelapati et al., 2022; Grégoire et al., 2007; Meng et al., 2016; Deardorff et al., 2012; Qin et al., 2024; Luo et al., 2019; Han et al., 2022; Wang et al., 2021; Zhu et al., 2011; Ye et al., 2025; Liu et al., 2024; Sanguigno et al., 2023; Tao et al., 2007; Zhang et al., 2023d; Song et al., 2024; Zheng et al., 2015; Li et al., 2024b; Fan et al., 2020; Lee et al., 2015; Liu et al., 2025; Li et al., 2018; Radhakrishnan et al., 2015; Yamagata and Kitabayashi, 2009; Vaquero et al., 2004; Xie et al., 2024; Menssen et al., 2012; Chen et al., 2022; Wu et al., 2022; Xu et al., 2016; Jing et al., 2007; Seo et al., 2015; Cai et al., 2023; North et al., 2003; Xu et al., 2025; Papa et al., 2014; Xian et al., 2025; Novgorodov et al., 2016; Wang et al., 2022; Li C. et al., 2023; Zhang F. et al., 2022; Nakagawa et al., 2009; Shi et al., 2019; Guo Z. et al., 2023; Geng et al., 2020; Zhang et al., 2014; Barber et al., 2012; Blank et al., 2017; Mo et al., 2017; Vakhrusheva et al., 2008; Ryu et al., 2014; Liu et al., 2009; Núñez-Álvarez and Suelves, 2022). Current classification frameworks delineate that these enzymatic families are systematically partitioned based on their characteristic catalytic and structural domains, with distinct KAT and KDAC subgroups evolving to regulate spatially distinct molecular targets. Remarkably, the scope of acetylation substrates has expanded beyond chromatin-associated proteins to encompass pivotal molecular effectors governing cellular fate determination, including but not limited to metabolic enzymes, transcription factors, DNA repair machinery components, and inflammatory mediators (Núñez-Álvarez and Suelves, 2022; Hishikawa et al., 2019; Ma et al., 2019). This substrate diversification underscores the central regulatory role of lysine acetylation in integrating diverse cellular signaling pathways, with profound implications for developing precision therapies targeting acetylation-dependent pathological mechanisms.

## 2 Physiological functions of acetylation/deacetylation modifications

**FIGURE 2:**
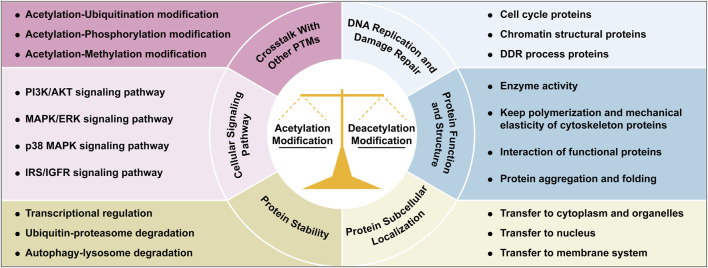
Integrated roles of acetylation/deacetylation in cellular regulation. This figure illustrates the multifaceted roles of acetylation and deacetylation modifications in coordinating cellular processes, through regulating genome homeostasis, protein function and activity, signaling pathways, protein dynamics and subcellular localization, and crosstalk with other PTMs. Each functional mechanism is illustrated with representative examples or key cellular processes. Abbreviations: AKT, protein kinase B; DDR, DNA damage repair; ERK, extracellular-signal-regulated kinases; IGFR, insulin like growth factor 1; IRS, insulin receptor substrate; MAPK, mitogen-activated protein kinases; PI3K, phosphatidylinositol 3-kinase; PTM, post-translational modification.

Acetylation and deacetylation maintain cellular homeostasis through a dynamic balance, regulating processes such as nucleic acid and protein dynamics, DNA replication/repair, protein synthesis/degradation, subcellular localization, and signaling pathways (Figure 2). Acetylation/deacetylation modification occurs throughout cellular processes.

### 2.1 DNA replication and damage repair

Acetylation and deacetylation maintain cellular homeostasis by dynamically regulating processes such as DNA replication, protein function, and signaling. HDACs and HATs interact with cell cycle regulators like pRb and Mad/Max to control progression; for example, HDAC-pRb suppresses cyclin-dependent kinase 2 (Cdk2) expression, while p300 acetylates cyclin D1 to drive the progression of G1 phase (Knoepfler and Eisenman, 1999; Nomura et al., 1999; Albanese et al., 1999; Resnitzky et al., 1994). During mitosis, HAT5 acetylates histones (H2A/H3/H4), and HAT2B/HAT5 modify Aurora B and EB1 to ensure accurate chromosome segregation (Mo et al., 2016; Ward et al., 2013; Xia et al., 2012). Structural maintenance of chromosomes 3 (SMC3) acetylation by ESCO1/2 stabilizes sister chromatid cohesion, and its deficiency disrupts DNA repair (Zhang et al., 2008; Rolef Ben-Shahar et al., 2008; Unal et al., 2008; Zhao et al., 2010). In DSB repair, TIP60 activates ATM *via* acetylation, while ATP-citrate lyase (ACLY)-derived nuclear acetyl-CoA reduces TP53-binding protein 1 (53BP1) recruitment, favoring homology-directed repair (HDR) over non-homologous end-joining (NHEJ) (Sun et al., 2005; Sivanand et al., 2017).

### 2.2 Protein function and structure

Acetylation modulates enzyme activity by targeting catalytic lysine residues: mitochondrial ACSS2 is inhibited by acetylation but reactivated by Sirtuin deacetylase family 3 (SIRT3) mediating deacetylation, whereas PGAM1 acetylation enhances glycolysis but is suppressed by SIRT1 under glucose starvation (Schwer et al., 2006; Hallows et al., 2006; Fujino et al., 2001; Walter et al., 1999). Autoacetylation of HATs like P300/CREB-binding protein (CBP) and MYST1 activates their catalytic domains. Structurally, α-tubulin acetylation (alpha-tubulin acetyltransferase 1 (ATAT1)-mediated) strengthens microtubule elasticity, while HDAC6 deacetylation regulates dynamics (Akella et al., 2010; Hubbert et al., 2002; Zhang et al., 2003). Pathologically, Tau acetylation at Lys174 promotes neurotoxic aggregates in Alzheimer’s disease, while acetylation at Lys259/Lys353 inhibits hyperphosphorylation-driven aggregation (Cohen et al., 2011; Min et al., 2010; Min et al., 2015; Cook et al., 2014).

### 2.3 Protein subcellular localization

Acetylation alters protein charge or interactions to dictate localization. S-phase kinase-associated protein 2 (SKP2) acetylation promotes cytoplasmic retention by blocking nuclear import, whereas hepatocyte nuclear factor 4 (HNF4) acetylation enhances nuclear retention (Frescas and Pagano, 2008; Inuzuka et al., 2012; Soutoglou et al., 2000; Zhao LJ. et al., 2006). Glyceraldehyde-3-phosphate dehydrogenase (GAPDH) acetylated by P300/CBP-associated factor (PCAF) translocates to the nucleus for DNA repair, and kinase suppressor of Ras-1 (CNK1) acetylation directs it to the plasma membrane to amplify RAS/mitogen-activated protein kinases (MAPK) signaling (Ventura et al., 2010; Fischer et al., 2017). Mitochondrial p66Shc acetylation increases reactive oxygen species (ROS) production by facilitating its mitochondrial translocation (Kumar et al., 2017).

### 2.4 Protein stability

Histone acetylation loosens chromatin to activate transcription, exemplified by Spt-Ada-Gcn5 acetyltransferase (SAGA) complex recruitment of RNA Pol II and bromodomain-containing protein 4 (BRD4) binding to acetylated H3/H4 (Cazzalini et al., 2014; Fischer et al., 2017; Li et al., 2008). In *Drosophila melanogaster*, HAT8-mediated H4K16 acetylation preserves epigenetic memory across generations (Samata et al., 2020; Tadros and Lipshitz, 2009). Acetylation competes with ubiquitination to regulate degradation: small mothers against decapentaplegic 7 (Smad7) acetylation blocks Smad ubiquitination regulatory factor 1 (Smurf1)-mediated ubiquitination, stabilizing it, while DNA (cytosine-5)-methyltransferase 1 (DNMT1) acetylation triggers ubiquitin-like with PHD and RING finger domains 1 (UHRF1)-dependent degradation (Du et al., 2010). Autophagy targets acetylated pyruvate kinase (PKM2) and lactate dehydrogenase A (LDHA) for lysosomal degradation, and SIRT1 deficiency impairs basal autophagy (Lv et al., 2011; Zhao et al., 2013; Lee et al., 2008).

### 2.5 Cellular signaling pathways

Cellular signaling networks are information-processing networks that control almost all cellular functions, and acetylation of key proteins in signaling pathways enable cells to respond rapidly to internal and external signals. Phosphatase and tensin homolog (PTEN) acetylation by PCAF inhibits phosphatase activity, whereas protein kinase B (AKT)/3-phosphoinositide-dependent kinase 1 (PDK1) acetylation blocks phosphatidylinositol (3,4,5)-triphosphate (PIP3)-dependent membrane activation (Okumura et al., 2006). CNK1 acetylation recruits rapidly accelerated fibrosarcoma kinase (RAF) to the membrane, amplifying extracellular-signal-regulated kinases (ERK) signaling (Fischer et al., 2017). SIRT1 deacetylates insulin receptor substrate 2 (IRS2) to enhance insulin signaling, and p38 autoacetylation boosts ATP binding during stress (Li et al., 2008) (Pillai et al., 2011). Tat-interacting protein 60 (TIP60) acetylates ataxia-telangiectasia mutated kinase (ATM) to activate DNA damage responses, while mitogen-activated protein kinase phosphatase 1 (MKP1) acetylation enhances phosphatase activity to suppress MAPK signaling (Sun et al., 2005; Pillai et al., 2011).

### 2.6 Crosstalk with other PTMs

Acetylation interacts with ubiquitination, phosphorylation, and methylation. For example, p53 acetylation by CBP/P300 competes with murine double minute 2 (MDM2)-mediated ubiquitination, stabilizing p53 (Li et al., 2002). In IFNα signaling, STAT2 acetylation facilitates IFN-stimulated gene factor 3 (ISGF3) complex formation, amplifying antiviral gene transcription (Tang et al., 2007). TGF-β signaling involves Smad7 acetylation (by P300) to block ubiquitination, whereas HDAC1 deacetylation promotes Smurf1-mediated degradation (Grönroos et al., 2002; Simonsson et al., 2005).

## 3 Acetylation modifications are associated with senescence

**FIGURE 3:**
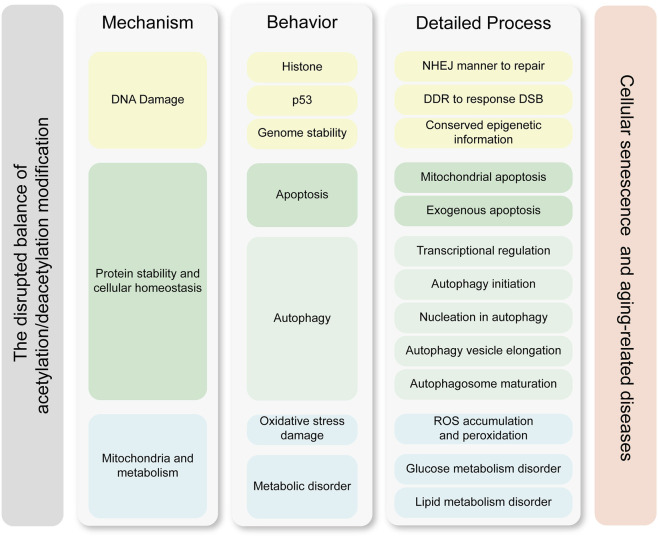
Molecular mechanisms linking acetylation dynamics to cellular senescence. This figure delineates the interplay between acetylation modifications and cellular senescence through three core mechanisms, which are DNA damage, protein stability and cellular homeostasis, and metabolic homeostasis. Key processes such as transcriptional regulation, genetic stability maintenance, and exogenous apoptosis further highlight how acetylation imbalances drive senescence-associated pathologies. The left column represents the core mechanisms by which acetylation modifications contribute to cellular senescence and age-related diseases. The middle column illustrates the key functional processes and effector molecules involved, while the right column details the specific molecular processes and pathways through which these effects are exerted. The mechanisms in the left column drive the process in the middle column, and the processes in the right column further refine the molecular pathways underlying these behaviors. The cellular processes and specific effective mechanisms are clustered by the similar color. Abbreviations: DDR, DNA damage repair; DSB, double strain break; NHEJ, non-homologous end-joining; ROS, reactive oxygen species.

Growing evidences suggest that acetylation is an important component of altering epigenetic marks during cellular senescence. Cellular senescence arises not from catastrophic loss of individual functional units or isolated molecular deficiency, but rather from dysregulation in multiscale biological processes. We believe the pathophysiological basis of aging manifests as dual-tiered coordination failures, which compromised homeostatic integration within cellular biochemical coordination and disrupted coordination across intercellular cross-talk. This hypothesis is substantiated by seminal work from Ana Carolina Leote et al., whose systematic analysis of aging transcriptomes revealed that while most gene-gene relationships in transcriptional regulatory networks remain preserved during aging, the deterioration of regulatory coupling predominantly stems from the loss of multiscale integration across cellular processes (Leote et al., 2024). These findings provide compelling validation for our hypothesis that senescence constitutes a systems-level failure of biological coordination rather than discrete component malfunction. Acetylation is a PTM that affects protein function regulating double strand break-DNA damage response (DSB-DDR), metabolic pathways and protein homeostasis. Hence, once acetylation modifications are disrupted, this balance is disrupted and eventually leads to cellular senescence (Kanfi et al., 2012; Levy et al., 2020). Figure 3 illustrates that an imbalance in acetylation-deacetylation homeostasis drives cellular senescence through three interconnected mechanisms, DNA damage accumulation, protein homeostasis disruption, and mitochondrial dysfunction.

### 3.1 DNA damage

Senescence is characterized by the loss of genomic integrity and stability, which involves chromatin remodeling, transcription dysregulation, and accumulation of DNA damage, as well as deficiencies in the ability to respond to DDR. HDAC1 plays a pivotal role in maintaining genomic integrity, supporting neuronal survival, and modulating synaptic plasticity, and collectively constituting a critical defense against neurodegenerative pathogenesis (Kim et al., 2008; Dobbin et al., 2013; Wang et al., 2013). Recruitment of HDAC1 by histones triggers hypoacetylation of H56K3 and H16K4, thereby promoting DSB repair through NHEJ, which is the major neuronal repair pathway (Miller et al., 2010; Madabhushi et al., 2014). p53 serves as a critical effector molecule in the DDR pathway following genotoxic stress (d'Adda di Fagagna, 2008). It governs cellular senescence and fate determination through mediating key processes including apoptosis and cell cycle arrest. Importantly, acetylation modifications have been established as a crucial regulatory mechanism linking p53 to senescence induction (Hong et al., 2010). Acetylated p53 attenuates the effect of Mdm2 on the ubiquitination degradation of p53 to enhance p53 stability, and further recruits HATs to the promoters of genes involved in the DDR process and cell cycle control mechanism, thereby mitigating DNA damage-induced senescence (Li et al., 2002; Ito et al., 2002). The SIRTs also participate in DDR processes; SIRT1, SIRT6, and SIRT7 have been shown to be directly involved in DSB repair. For example, SIRT7 was recruited to damage sites of DNA in a PARP-1 dependent manner and regulated H3K18 acetylation at the damaged sites. Acetylated H3K18 in turn affected the recruitment of the damage response factor 53BP1 to the damaged DNA sites, which ultimately enhanced the efficiency of NHEJ and promoted DNA damage repair. Correspondingly, the depletion of SIRT7 led to a decline in the repair capacity of DSBs (Vazquez et al., 2016).

In addition, the accumulation of mutant DNA during the genetic process has been found to be a major cause of senescence, while some studies indicated that mice or patients with higher mutation rates did not exhibit a premature aging phenotype. However, senescent mammalian cells still had the ability to clone new individuals with a normal life expectancy, suggesting there might be a mechanism beyond mutation-induced senescence that linked DNA stability and cellular senescence (Burgstaller and Brem, 2017; Robinson et al., 2021; De Majo et al., 2021). An increasing number of studies have shown that senescent cells have a deficiency of genetic information and a loss of epigenetic information. Some researchers believe that the epigenetic alterations caused by DNA damage that induce the relocation of chromatin modifying factors might be the major cause of senescence, and further proposed a hypothesis in which the loss of epigenetic information is the cause of senescence (Yang et al., 2023; Lu et al., 2020; Pan et al., 2023). DSBs are one of the major factors that cause time-varying epigenetic changes, and the epigenetic regulators SIRT2 and general control non-depressible 5 (GCN5) are required in the DDR process to repair DSBs, such as mediating acetylation of histones involved in aging. Senescent cells exhibit a higher acetylation level of H3K122 and lower acetylation level of H3K27. H3K27ac is usually enriched in accessible regions, including promoters and enhancers, but the highly accessible regions of senescent cells lose their original H3K27 acetylation, while less accessible regions have increased levels of H3K27 acetylation. This DSB-induced epigenomic change accelerates the production of age-related markers, and epigenetic remodeling can reverse these changes and restore the cellular epigenome to a youthful state (Yang et al., 2023).

ACLY is the important enzyme that catalyzes the reaction which products acetyl-CoA. ACLY and histone acetylation also play critical roles in regulating chromatin structure and gene expression. ACLY converts citrate derived from glucose metabolism into acetyl-CoA, the primary substrate for histone acetylation, thus directly influencing global histone acetylation levels (Wellen et al., 2009). During the DDR, ACLY is phosphorylated at Ser455 by ATM and AKT, leading to its nuclear localization (Sivanand et al., 2017). This promotes histone acetylation near DNA damage sites, such as H3K9ac and H4K16ac, which inhibits 53BP1 binding while enhancing BRCA1 recruitment, thereby driving homologous recombination (HR) repair (Sivanand et al., 2017). SIRT6, an NAD+ -dependent deacetylase, regulates chromatin stability and repair through deacetylation during DNA damage and aging, playing a central role in DDR and senescence (Onn et al., 2020). Age-related decline in NAD + levels may impair SIRT6 activity, resulting in accumulated histone acetylation and compromised repair capacity, exacerbating genomic instability. Upon DNA damage, studies demonstrate that SIRT6 directly recognizes and binds to DSBs *via* its core structural domain, acting as an independent damage sensor to activate downstream repair pathways, such as ATM recruitment and H2A histone family member X (H2AX) phosphorylation, and recruit repair proteins such as breast cancer type 1 susceptibility protein (BRCA1) and 53BP1 (Sivanand et al., 2017; Onn et al., 2020). The deacetylase activity of SIRT6 is essential for chromatin remodeling and DNA repair. Its deficiency leads to genomic instability, telomere dysfunction, and accelerated aging phenotypes (Onn et al., 2020). SIRT6 suppresses ACLY-mediated histone acetylation by removing acetyl groups from histone H3K9 and facilitates DNA-DNA dependent protein kinase catalytic subunit (PKcs) localization on chromatin to enhance DNA repair (McCord et al., 2009). The synergistic interaction between ACLY and SIRT6 (dynamic regulation of acetylation and deacetylation) likely holds significant implications for maintaining genomic integrity and delaying aging (Sivanand et al., 2017; Wellen et al., 2009; Onn et al., 2020; McCord et al., 2009).

### 3.2 Protein stability and cellular homeostasis

#### 3.2.1 Apoptosis

Apoptosis is involved in all stages of development and senescence, and there is growing evidence that senescence and apoptosis are distinct cell fate outcomes that interact with each other (Basu, 2022; Childs et al., 2014; Schmitt, 2003). Aberrant apoptosis affects whether cells go into senescence or not, making apoptosis a potential mechanism to regulate senescence. The SIRTs affect various apoptotic regulation factors by regulating acetylation modifications. SIRT1, SIRT6, and SIRT7 mitigate p53-dependent apoptosis through deacetylation modifications. SIRT7, for example, was shown to enhance cardiomyocyte stress resistance and ultimately inhibit apoptosis by deacetylating p53, and SIRT7-deficient mice exhibited an elevated apoptotic response and severe cardiac inflammatory cardiomyopathy phenotype (Vakhrusheva et al., 2008; Zeng et al., 2022; Vaziri et al., 2001; Wood et al., 2018; Yao et al., 2018). SIRT1, SIRT2, and SIRT3 deacetylate and activate forkhead box-O-3a (FoxO3a) to promote the activation of the c-Jun N-terminal kinase (JNK) signaling pathway, which ultimately increased apoptosis (Yao et al., 2018; She et al., 2018; Peng et al., 2019). SIRTs also regulate the molecules that directly participate the apoptosis process. For example, SIRT5 deacetylated cytochrome C and reduced cytochrome C leakage to further alleviate endogenous apoptosis (Schlicker et al., 2008; Yang et al., 2021; Li et al., 2019).

#### 3.2.2 Autophagy

Dysfunction of autophagy induces senescence and can promote aging phenotypes, and biochemical intervention or genetic manipulation to regulate autophagy can partially delay the aging process (Franco-Juárez et al., 2022; Goehe et al., 2012). Acetylation modifications are involved in the entire process of autophagy, including transcriptional regulation of genes in the initiation stage of autophagy to lysosomal degradation as the outcome of autophagy behavior (Sun et al., 2021). In terms of the transcriptional stage, activated transcription factor EB (TFEB) is an important factor in the transcriptional regulation of autophagy. Activated TFEB binds to CLEAR motifs to regulate the transcription of autophagy-lysosome related genes, promoting autophagic biogenesis and lysosomal fusion to increase autophagy, thereby effectively degrading molecular complex and preventing induced aging. ATAT1 mediated Lys91, Lys103, and Lys430 acetylation of TFEB to promote a transcriptional effect, while the HAT2/GCN5 mediated Lys116, Lys274, and Lys279 acetylation disrupted the dimerization of TFEB and decreased binding to the promoter of its target genes, indicating that the acetylation mode of TFEB directly affected transcriptional activity (Wang et al., 2020). The HAT8/SIRT1 system, which mediates H4K16 acetylation, suppresses the transcription of early-stage and end-stage autophagy related genes (Salminen and Kaarniranta, 2009; Füllgrabe et al., 2013). SIRT1 was also shown to indirectly regulate autophagy by upregulating the expression of the autophagy-related gene Bnip3 through the deacetylation of FoxO3 (Kume et al., 2010; Kroemer et al., 2010).

In the autophagy initiation process, starvation stimulation activates the glycogen synthase kinase 3β (GSK3β)/TIP60/UNC-52-like kinase 1 (ULK1) pathway to regulate autophagy behavior. GSK3β phosphorylates TIP60-Ser86 to an activated state, and the activated TIP60 further acetylates ULK1 on Lys162 and Lys606 sites to activate downstream protein kinases, subsequently initiating autophagy through the mechanistic target of rapamycin (mTOR) signaling pathways (Lin et al., 2012; Dossou and Basu, 2019; Nie et al., 2016). In the following autophagic lysosome formation process, P300-mediated acetylation controls the activity of the phosphatidylinositol 3-kinase catalytic subunit type 3/vacuolar protein sorting 34 (PIK3C3/VPS34) signaling pathway, which forms a complex with Beclin1 and further directly regulates autophagy nucleation (Su et al., 2017; Kary, 2018; Su and Liu, 2018). Furthermore, acetylated tripartite motif proteins (TRIMs) or heat shock protein 70 (Hsp70) indirectly affected autophagy by regulating other PTMs of key proteins in the autophagy nucleation process (Yang et al., 2013; Fusco et al., 2018). The P300/SIRT1 system is the key regulator of acetylation modifications during autophagic vesicle extension and the LC3 conjugation process (Liu and Klionsky, 2015; Sebti et al., 2014). In the last process, acetylated Beclin1 and TIP60 mediates the acetylation of downstream molecules to regulate autophagosome maturation through the RUN domain and Cysteine-rich domain containing, Beclin 1-interacting protein (RUBCN) bimolecular switch model (Matsunaga et al., 2009; Zhong et al., 2009; Cheng and Sun, 2019; Cheng et al., 2019). The reversible acetylation of α-microtubule proteins and HDAC6/SIRT1-mediated acetylation of cortactin protein is required for autophagosome–lysosome fusion (Lee et al., 2010; Zhang et al., 2009; Köchl et al., 2006).

### 3.3 Mitochondria and metabolism

Extensive experimental evidence demonstrates that mitochondrial dysfunction leads to aging processes, as mitochondria critically influence or regulate multiple key aspects of senescence (Sun et al., 2016). Notably, mitochondria-targeted interventions and strategies improving mitochondrial quality and function may exert profound beneficial effects, effectively mitigating the deleterious impact of senescence in aging tissues by restoring redox homeostasis and bioenergetic capacity (Sun et al., 2016; Correia-Melo et al., 2016).

#### 3.3.1 Oxidative stress damage

In 1972, Harman synthesized the seminal proposal, which is defined as mitochondrial free radical theory of aging, that aging stems from cumulative free radical-induced damage to mitochondria. This hypothesis links the mitochondrial homeostasis and aging, which posits an age-associated elevation in mitochondrial ROS generation, wherein ROS accumulation inflicts oxidative lesions across critical biomolecules, such as mitochondrial DNA (mtDNA) mutations, lipid peroxidation, protein carbonyl formation and nucleic acid modifications (Harman, 1972). These molecular injuries propagate cellular dysfunction through compromised electron transport chain efficiency, impaired calcium homeostasis, and defective mitochondrial quality control mechanisms, ultimately manifesting as multi-tiered physiological decline across tissues and organ systems (Miwa et al., 2022; Guo et al., 2023a). Oxidative stress damage is one of the important factors of mitochondria-induced senescence, in which the oxidative stress-induced abnormal accumulation of ROS exceeds the cell’s scavenging capacity, leading to disrupted intracellular homeostasis and senescence. Increasing evidence has indicated that SIRTs regulate oxidative stress damage by deacetylation modification of various crucial factors involved in metabolism regulation, inflammation, and antioxidant responses. The most common SIRTs that participate in the antioxidant responses are SIRT1, SIRT3, and SIRT6.

AMP-activated protein kinase (AMPK) is a crucial kinase in the energy regulation process and is recognized as a core regulatory factor of eukaryotic cells. SIRT1 and SIRT3 were found to deacetylate live kinase B1 (LKB1) which is the upstream regulator of AMPK, and deacetylated LKB1 then activated AMPK to reduce ROS accumulation and lipid peroxidation (Li et al., 2020). SIRT6 directly interacted with AMPK and promoted the expression of antioxidant stress proteins, such as superoxide dismutase (MnSOD) and human catalase targeted to the mitochondria (mCAT), which inhibited oxidative stress damage (Wang et al., 2016). A similar mechanism was also observed in SIRT1-and SIRT3-mediated FoxO deacetylation that performed antioxidant function (Sundaresan et al., 2009; Kwon et al., 2015; Yang et al., 2016). SIRT1/2/6 regulated the transcriptional activity of nuclear factor erythroid 2-related factor 2 (Nrf2) by a deacetylation modification to protect cells from ROS damage (Chen et al., 2020; Zhao et al., 2021; Yu et al., 2019).

#### 3.3.2 Glucose metabolism and lipid metabolism

Protein acetylation modifications have emerged as a major mechanism for regulating cellular metabolism. Phosphoenolpyruvate carboxy-kinase (PEPCK1) is the key enzyme and rate-limiting enzyme that catalyzes the synthesis of glucose from pyruvate. An acetylation modification regulates the stability of PEPCK1 to affect the balance between glucose metabolism and generation, thus achieving glucose homeostasis *in vivo*. When the glucose concentration increases, P300 mediates the acetylation of PEPCK1 and promotes interaction with the E3 ubiquitin ligase UBR5, facilitating further PEPCK1 ubiquitination and proteasome degradation, thereby limiting glucose production. SIRT1 also improves the glucose metabolism status and maintains energy homeostasis, such as by upregulating and activating AMPK (Peng et al., 2010; Silvestre et al., 2014). SIRT1 was also shown to downregulate the activity of protein tyrosine phosphatase 1B (PT1B), which is a key negative regulator of the insulin signaling pathway, thereby improving insulin sensitivity and reducing serum glucose (Gouranton et al., 2014). Another deacetylase with a substantial impact on metabolism is SIRT6. As the deacetylase of H3K9, SIRT6 inhibits the expression of several glycolytic genes (Zhong et al., 2010). SIRT6 also deacetylates FoxO1, which in turn increases the transcription and expression of glucose-dependent transporter 2 to maintain the glucose-sensitizing capacity of pancreatic islet β-cells and glucose tolerance, delaying high-glucose–induced chronic inflammation and senescence (Song et al., 2016).

Increasing evidence confirmed that Acetyl-CoA-induced acetylation modifications led to abnormal accumulation of fatty acids and eventually vascular aging (Lin et al., 2022). The SIRTs have been implicated as the major factors in the retardation of senescence in deacetylation modifications. The main target of SIRT1 are FoxOs, which act as important transcriptional regulators, and deacetylation of FoxOs promotes the transcription of fatty acid oxidation-related proteins (Mostoslavsky et al., 2006). SIRT3 plays a bridging role in lipid metabolic pathways and regulates mitochondrial acetylation levels. SIRT3 has been demonstrated to be involved in almost all processes of mitochondrial metabolism and homeostasis (Samant et al., 2014; Papa and Germain, 2017), such as deacetylating and enhancing isocitrate dehydrogenase 2 (IDH2) and long-chain acyl-CoA dehydrogenase (LCAD) activities to stimulate fatty acid β-oxidation and mitigate cellular senescence.

## 4 Acetylation modifications are associated with aging-related diseases

**FIGURE 4:**
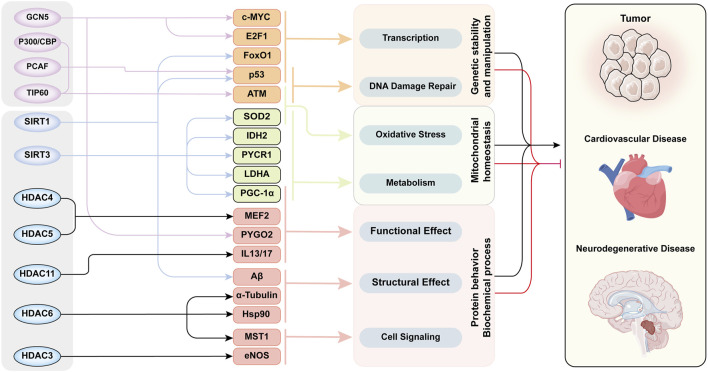
The Acetylation modification -Network mechanism -Senescence mode in aging-related diseases. This figure exhibited functional network of acetylation/deacetylation in cellular processes and disease pathogenesis. Multiple key proteins are acetylated by several HATs/HDACs to induce senescence through various cellular processes and biochemical reactions. The functions of these proteins intersected with each other and ultimately led to aging through three aspects of cellular behavior. The whole acetylation-related regulatory mechanism form a cross network to regulate the development of aging-related diseases. This diagram categorizes histone acetyltransferases and deacetylases in the leftmost column, their substrate targets in the second column, the associated cellular processes in the third column, and linked diseases in the rightmost column. Substrates are color-clustered by functional categories. Black lines between the third and fourth columns denote disease-promoting effects, while red lines indicate inhibitory roles. Abbreviations: ATM, ataxia-telangiectasia mutated kinase; E2F1, E2 promoter binding factor 1; eNOS, endothelial nitric oxide synthase; FoxO1, Forkhead box-O-1; GCN5, general control non-depressible 5; HDAC, histone deacetylase; Hsp90, heat shock protein 70; IDH2, isocitrate dehydrogenase 2; IL13/17, interleukin-13/17; LDHA, lactate dehydrogenase A; MEF2, myocyte enhancer factor 2; MST1, mammalian sterile 20-like kinase 1; P300/CBP, P300/CREB-binding protein; PCAF, P300/CBP-associated factor; PGC-1α, Peroxisome proliferator-activated receptor-gamma co-activator-1alpha; PYCR1, pyrroline-5-carboxylate reductase 1; PYGO2, Pygopus 2; SIRT, Sirtuins family; SOD2, superoxide dismutase 2; TIP60, Tat-interacting protein 60.

In addition to natural aging, cellular senescence is observed in various aging-related chronic diseases, such as tumors, hypertension, and neurodegenerative diseases. Cellular senescence is also an important factor accelerating disease progression, and is recognized as a common end-stage pathological condition of many diseases. Figure 4 illustrates that acetylation modifications orchestrate the pathogenesis of aging-related diseases through interconnected regulatory networks, dynamically coordinating DNA damage repair, protein homeostasis, and mitochondrial integrity.

### 4.1 Tumors

Senescent cells represent a deficiency of genomic stability, which leads to an increasing probability of various DNA mutations and excessive accumulation of abnormal DNA, activating the expression of proto-oncogenes and ultimately promoting cellular carcinogenesis. Therefore, most tumors are considered to be aging-associated diseases. Acetylation modifications activate the enhancer of H4K16, further regulating chromatin structure and transcriptional regulation. whereas the loss of H4K16ac is considered to be a universal marker for the development and malignant transformation of tumors (Taylor et al., 2013; Fraga et al., 2005; Seligson et al., 2005). Changes in histone acetylation patterns can also be used to predict the prognosis and recurrence of cancer. For example, the insufficient acetylation of H3K9, H3K14, and H4K12 were strongly associated with an adverse prognosis of breast cancer, and low acetylated levels of H3K9, H3K18, and H4K16 were shown to be important markers for prostate cancer recurrence (Seligson et al., 2005). The acetylation of non-histone proteins is also involved in a variety of crucial life processes, such as DNA replication, metabolism, differentiation, and development, and abnormal acetylation modifications often disrupt the balance of these cellular processes, eventually leading to cancer (Glozak et al., 2005; Singh et al., 2010; Spange et al., 2009).

#### 4.1.1 HATs in tumorigenesis

A large number of transcription factors are targeted substrates of HATs, including oncogenes and tumor suppressor genes associated with tumorigenesis, such as *MYC a*nd *TP53*. Myc protein, which is encoded by the gene *c-MYC*, is one of the most abundant oncogene proteins in cancer, and is stabilized by GCN5 with an acetylation modification at Lys323 (Patel et al., 2004). GCN5 can also be recruited by Myc to the template of Pol III, resulting in a significant increase in histone H3 acetylation, and ultimately promoting the transcription of *c-MYC* target genes (Kenneth et al., 2007). HAT2A regulated other transcription factors or tumor-promoting proteins, such as E2 promoter binding factor 1 (E2F1) and pygopus2 (PYGO2), to enhance the expression of target oncogenes in a similar manner, most of which stimulated abnormal cell proliferation and tumor growth (Chen et al., 2013; Chen et al., 2010). However, acetylation modifications also regulate the expression or function of tumor suppressor genes. Several HATs mediate tumor suppressor p53 acetylation modifications to increase the stability of p53 and enhance binding with DNA. CBP/P300 acetylates the carboxyl terminus of p53, preventing its proteasomal degradation (Li et al., 2002). TIP60 plays an important role in maintaining integrity of DNA and preventing the accumulation of tumor-inducing mutations. Under DSB conditions, acetylation and activation of the kinase ATM by TIP60 is the key step for subsequent recruitment of proteins involved in the DNA repair process that includes p53 (Sun et al., 2010).

#### 4.1.2 HDACs in tumorigenesis

Different deacetylases cause various effects on the development of tumors by deacetylating different substrates, which drives tumor cells to different destinies. HDAC family-mediated deacetylation modifications are often observed in tumor progression and deterioration. HDAC6-mediated deacetylation of mammalian sterile 20-like kinase 1 (MST1) decreased protein stability and promoted the aggravation of breast cancer (Li et al., 2016). HDAC6 also promoted cancer development by deacetylating a-tubulin to enable cells to metastasize through malignant migration, chemotaxis, and angiogenesis (Valenzuela-Fernández et al., 2008; Kaluza et al., 2011). Hsp90 is another important substrate of HDAC6 which is activated after deacetylation modification. The activated Hsp90 further stabilized and promoted the transcription of the androgen receptor, eventually inducing the development of prostate cancer (Gao and Alumkal, 2010). We also noticed that HDACs negatively regulated the process of cancer and improved the prognosis. HDAC10 attenuated lymph node metastasis of cervical cancer by deacetylating the histones H3 and H4, which downregulated the expression of matrix metalloproteinase 2/9 (MMP2/9), thus mitigating migration and invasion (Song et al., 2013).

The SIRTs mainly mediate deacetylation of non-histone proteins and regulate cell behavior involving multiple tumorigenesis process, and cause different regulatory directions in tumor cells. SIRT1 is believed to be a factor in tumorigenesis and has been observed to be highly expressed in a variety of cancers, including prostate cancer and acute myeloid leukemia. SIRT1 was shown to bind and promote FoxO1 to translocate into the nucleus, enhancing the transcription of target genes involved in cancer development (Bosch-Presegué and Vaquero, 2011). SIRT1 also deacetylates c-Myc and further negatively regulates the transcription of tumor suppressor-related genes to promote the development of cancer (Yuan et al., 2009). Some studies observed downregulation of SIRT2 in glioma and gastric cancer, suggesting that SIRT2 might play a suppressive effect in cancer development (Inoue et al., 2007). SIRT3 regulates mitochondrial metabolism through deacetylation modifications and participates in cellular metabolism reprogramming, which plays a unique function by mediating the interaction between mitochondria and intracellular signal transduction, and is considered to be a hallmark molecule of cancer. SIRT3 plays different roles in various cancers and is specific to tumor type (Ouyang et al., 2022). SIRT3 regulated IDH2 dimerization by mediating Lys413 site acetylation of IDH2, which regulated the metabolism and progression of breast cancer (Zou et al., 2017). SIRT3 deacetylated the Lys228 site of pyrroline-5-carboxylate reductase 1 (PYCR1) to regulate the metabolism of proline, which promoted the development of breast cancer (Chen et al., 2019). In addition, a study revealed that SIRT3 participated in the mitochondrial metabolic balance of gastric cancer and promoted deacetylation and activation of LDHA, thereby enhancing glycolysis and proliferation of gastric cancer, and was considered a cancer promoting factor (Cui et al., 2015). Some studies focused on colon cancer have demonstrated that SIRT3 was highly expressed in colorectal cancer and was associated with its tumor stage and lymph node metastasis (Liu et al., 2014). SIRT3 has been confirmed to enhance chemotherapy resistance of colon cancer by modulating the acetylation of superoxide dismutase 2 (SOD2) and peroxisome proliferator-activated receptor-gamma co-activator-1alpha (PGC-1α), which enabled SIRT3 to be an independent prognostic factor for colon cancer (Paku et al., 2021).

#### 4.1.3 HDAC inhibitors in tumorigenesis

HDACs inhibitors induce cell cycle arrest, apoptosis, and necrosis in cancer cells. Some studies demonstrated that HDAC inhibitors enhanced the acetylation level and prolonged the half-life of p53, thereby increasing the interaction with the promoter of *p21* (Gius et al., 2004; Zhao Y. et al., 2006; Richon et al., 2000). Additionally, HDAC competed with p53 to bind to p21, leading to decreased transcription of p21 (Ocker and Schneider-Stock, 2007; Mahyar-Roemer and Roemer, 2001). Therefore, HDAC inhibitors increase p21 expression by this mechanism, which blocks the dimerization of cell cycle proteins and arrests the cellular cycle, eventually inhibiting the proliferation of tumor cells (Richon et al., 2000; Sandor et al., 2000). HDAC inhibitors also induce endogenous apoptosis in tumor cells by regulating the transcription of pro-apoptotic or anti-apoptotic genes, inhibiting the development and progression of tumor cells (Kim and Bae, 2011; Minucci and Pelicci, 2006; Zhu et al., 2004). HDAC11 negatively regulated the expression of interleukin-13/17 (IL-13/17) and tumor necrosis factor-alpha (TNF-α) in Hodgkin lymphoma, suggesting that HDAC11 inhibitors killed tumor cells by activating inflammatory responses, immune system activity, and apoptosis processes (Buglio et al., 2011).

### 4.2 Cardiovascular diseases

Clinical and molecular studies have observed significant alterations in the expression levels of multiple HDAC and SIRT proteins in pulmonary arterial hypertension patients, which demonstrated the potential and strong association between acetylation modifications and pathological vascular remodeling processes, such as proliferation, inflammation, and fibrosis (Chelladurai et al., 2021).

#### 4.2.1 Hypertension

Hypertension is a major factor in cardiovascular disease, and is mainly related to the activation of the renin-angiotensin-aldosterone system and sympathetic nervous system. Vascular injury is an important pathway for hypertension-induced target organ damage and multiple subsequent complications. The detailed pathological features include vascular smooth muscle cells (VSMCs) remodeling, mitochondrial dysfunction, and endothelial cell injury. It has been confirmed that HDAC4 and HDAC5 participate in Angiotensin II (Ang II)-induced hypertrophy of VSMCs, which led to vascular remodeling. Calmodulin kinase II promoted myocyte enhancer factor 2 (MEF2) activity through phosphorylation of HDAC4, thereby causing hypertrophy of VSMCs as well (Xu et al., 2007; Li et al., 2010). SIRT3-mediated deacetylation is essential for guaranteeing the activity of SOD2 to protect against vascular oxidative stress, and SIRT3 deficiency increased the production of mitochondrial superoxide free radicals and promoted the occurrence of hypertension (Dikalova et al., 2017). Endothelial nitric oxide synthase (eNOS) is the main producer of NO in vascular endothelial cells and is the core regulator of cardiovascular homeostasis; insufficient eNOS activity is a major pathogenic factor for endothelial dysfunction and hypertension. SIRT1 promotes endothelium-dependent vascular dilation by targeting eNOS for deacetylation and increasing its activity (Mattagajasingh et al., 2007). The vascular protective effect of aspirin may also be related to acetylation of eNOS (Jung et al., 2010), in which low-dose aspirin increased the vascular bioavailability of NO and promoted the binding of eNOS and calmodulin. In contrast, overexpression of HDAC3 inhibited aspirin-induced acetylation of eNOS, further antagonizing the effects of aspirin on NO production in endothelial cells (Jung et al., 2010).

#### 4.2.2 Atherosclerosis

Atherosclerosis is recognized as the most common disease affecting the vascular system, especially the coronary artery. The pathological feature is lipid accumulation in the smooth muscle layer and vessel blockage, and the resulting narrowed arteries, atherosclerosis, and unstable plaques may lead to the occurrence of myocardial infarction (Libby et al., 2011). VSMCs are the main cellular component in the artery, and epigenetic modification of VSMCs has been shown to contribute to the formation of atherosclerotic lesions (Hai and Zuo, 2016). Research has demonstrated that SIRT1 deficiency caused over-acetylation of eNOS, resulting in functional inactivation of eNOS, which further promoted the development of atherosclerotic plaques in mice, while HDAC3-mediated Lys610 deacetylation of eNOS promoted the atherosclerosis process (Jung et al., 2010; Yang et al., 2017; Rössig et al., 2002; Schermuly et al., 2011). Cellular senescence is another cause of atherosclerosis, and causes cells to be more susceptible to damage and further develop to atherosclerosis. La-related protein 7 (LARP7) is an aging antagonist molecule, which was downregulated by DNA damage-induced activation of ATM, and thereby inhibited the deacetylase activity of SIRT1. The ATM-LARP7-SIRT1-p53/p65 aging axis, which is composed of these molecules, is actively involved in the process of vascular aging and atherosclerosis, and blocking its activation significantly alleviated atherosclerosis (Yan et al., 2021).

### 4.3 Neurodegenerative disease

The accumulation of senescent cells in the central nervous system (CNS) serves as a core driver of cognitive decline and neurodegenerative diseases such as Alzheimer’s disease (AD) and Parkinson’s disease (PD) (Melo Dos Santos et al., 2024). The senescent nervous cells face insufficient energy substrate supply due to impaired local microcirculation, which fundamentally disrupts glial cell metabolism and ultimately damages neural networks. Altered energy metabolism in glial cells and its impact on brain aging and related disorders are currently a major research focus (Mattson and Arumugam, 2018). For example, cellular senescence promotes amyloidogenic cleavage of amyloid precursor protein (APP) and Tau pathology, while the accumulation of Aβ and Tau further amplifies senescence marker expression. Concurrently, lipid peroxidation-modified proteins accumulate in the aging brain, enhancing γ-secretase-mediated APP cleavage and inducing the production of neurotoxic Aβ42. These vicious cycles exacerbate AD progression through reciprocal reinforcement of senescence and neurodegenerative pathways. Neurological degeneration is another important feature of aging, in which HAT- and HDAC-regulated acetylation modifications play an indispensable role. An imbalance of acetylation modifications can lead to progressive neuronal-specific loss, impaired neuronal function, and ultimately neuronal death. Increasing evidence has shown that abnormal acetylation and deacetylation were associated with the pathogenesis of various neurodegenerative diseases, especially AD (Saha and Pahan, 2006).

During the development of AD, P300-mediated acetylation of histone H3, located in the promoter region of presenilin-1 (PS1) and beta-site amyloid precursor protein cleaving enzyme 1 (BACE1) aggravated neuronal injury, whereas targeted inhibition of P300 mitigated AD symptoms and abnormal protein aggregation. This suggested that P300 plays a key role in controlling the expression of AD-associated genes by regulating the acetylation of their promoter region (Lu et al., 2014). The stability of DNA replication guaranteed by HDAC1 has been found to play an important role in nervous system damage. The p25/Cdk5 complex involved in the AD process inactivated HDAC1, which led to aberrant cell cycle activity, double-stranded DNA breaks, and ultimately neurotoxicity, which was alleviated by the exogenous introduction of HDAC1 (Kim et al., 2008). In terms of molecular therapy for AD, SIRTs have been shown to regulate the progression of various neurodegenerative diseases by modulating transcription factor activity and protein toxicity (Gomes et al., 2019; Herskovits and Guarente, 2013). Abundant expression of SIRT1 inhibited Aβ polymerization and attenuated plaque formation, resulting in amelioration of behavioral deficits, suggesting a neuroprotective role for SIRT1 in AD (Jęśko et al., 2017). Small molecule SIRT activators, especially SIRT1 and SIRT2, have great potential in the treatment of age-related diseases, particularly neurodegenerative diseases (Han, 2009). They inhibit acetylation levels of various key process proteins and reduce toxic protein aggregates to restore protein homeostasis, and also enhance neuronal plasticity by promoting transcription of important genes responsible for memory and learning (Min et al., 2013). PD is defined as the most common neurological degeneration disease, especially in the geriatric population. α-Synuclein is an important effector of PD, which directly binds to histone H3 to mediate neurotoxicity. Various studies have confirmed that the inhibition of several HATs, such as P300/CBP and PCAF, reduced the level of H3 acetylation and alleviated α-synuclein-mediated nervous system damage, which has been verified in animal models. This provides support for histone deacetylase inhibitors as a potential treatment for PD (Kontopoulos et al., 2006).

## 5 Conclusions: “Acetylation-network regulation-cellular senescence” model

Based on the above evidence, we provide insight into the senescence mechanism and propose the concept that acetylation modifications play a pivotal effect in the senescence process. Macroscopically, acetylation modifications participate in the aging process through both direct and indirect pathways. Acetylation modifications play a pivotal role in orchestrating core cellular processes during senescence, encompassing the homeostasis and regulation of DNA maintenance and DDR process, protein stability and proteostasis, metabolic reprogramming and mitochondrial function. Acetylation modifications of histones ensure the stability of the DNA structure, and the acetylation of numerous DNA damage repair factors guarantees the integrity and accuracy of the DNA replication process. Acetylation modifications also occur on multiple transcription factors to regulate their transcriptional ability, altering the expression level of downstream target molecules. Acetylation modifications also directly regulate the activity, structure, or sub-location of various key proteins in senescent mechanisms, thereby affecting cellular fate through these senescence execution molecules. At the organelle level, mitochondria are the regulatory pivot of several cellular processes, and acetylation modifications regulate the activity of metabolism-related proteins to affect the metabolic function of mitochondria. At the organellar level, mitochondria act as a pivotal regulator of multifaceted cellular processes. Acetylation modifications orchestrate mitochondrial metabolic functions by dynamically modulating the activity of metabolism-associated proteins. Beyond metabolic regulation, acetylation modification critically coordinates non-metabolic pathways, including mitochondrial apoptosis and mitophagy. Such dual-axis regulation (metabolic/non-metabolic) ultimately governs cellular fate determination and senescence by integrating energy status with stress-responsive signaling. We believe that senescence likely operates through a networked model of interconnected and coordinated mechanisms. Upon exposure to endogenous damage or exogenous stressors, key nodes within this regulatory network simultaneously activate multiple pathways, including metabolic reprogramming, proteostasis regulation, genomic surveillance systems, and cell fate-determining signaling cascades, which collectively drive senescence through synergistic interactions. This model establishes an activation threshold requiring concurrent engagement of multiple pathways to reach critical signaling intensity, rather than relying on singular pathway activation. Crucially, this architecture prevents premature senescence commitment due to isolated pathway activation or single homeostatic imbalance. As a terminal cell fate, this multilayered control mechanism provides survival protective benefits by ensuring senescence occurs only when cumulative damage surpasses cellular adaptive capacity, thereby avoiding inappropriate cell cycle arrest under subcritical stress conditions.

Our proposed regulatory mode of acetylation modifications in senescence is a broad regulation of multiple key proteins in the senescence mechanism network. This mode regulates various downstream detailed senescence-related mechanisms at the same time, which is different from the classical mechanism of a single executive molecule affecting the terminal executors. We believe that the appropriate intervention of acetylation modifications is to move the intervention process forward, and intervene in multiple pathways at the same time through a mechanism network that does not rely on a single downstream senescence execution molecule. Acetylation modifications function as regulatory modalities rather than direct causative factors of senescence. These post-translational adjustments exert precise control over primary senescence-triggering processes, including metabolism, genomic homeostasis, mitochondrial function, and proteostasis networks, thereby indirectly modulating senescence-associated biological processes. This regulatory architecture explains the remarkable efficacy of acetylation-targeted interventions (e.g., chemical agents mimic HAT/HDAC activity) in ameliorating or even reversing senescent phenotypes, as they fundamentally recalibrate core aging mechanism through upstream pathway modulation. As a novel insight into the aging mechanism, the imbalance of cellular homeostasis caused by the disrupted acetylation modification balance systematically and comprehensively explains the changes of different cellular processes during senescence. For example, simple DNA damage might not lead to aging necessarily, while the reduced stability of the DNA-histone complex and the decreased activity of damage repair-related proteins caused by abnormal acetylation will cooperate to inevitably cause senescence. Therefore, the targeted intervention of the acetylation modifications of key nexus proteins or upstream regulators in the network mechanism is a new idea and direction for delaying or even reversing aging.

## Glossary

ACLY: ATP-citrate lyase
ACSS2: acetate-dependent acetyl-CoA synthetase 2
AMPK: AMP-activated protein kinase
APP: amyloid precursor protein
ATM: ataxia-telangiectasia mutated kinase
ATAT1: alpha-tubulin acetyltransferase 1
BACE1: beta-site amyloid precursor protein cleaving enzyme 1
BRCA1: breast cancer type 1 susceptibility protein
BRD4: bromodomain-containing protein 4
CBP: CREB-binding protein
Cdk2: cyclin-dependent kinase 2
CNS: central nervous system
CNK1: kinase suppressor of Ras-1
DNMT1: DNA (cytosine-5)-Methyltransferase 1
DDR: DNA damage response
DSB: double strand break
E2F1: E2 promoter binding factor 1
eNOS: Endothelial nitric oxide synthase
ERK: extracellular-signal-regulated kinases
FoxO3a: forkhead box-O-3a
GAPDH: Glyceraldehyde-3-phosphate dehydrogenase
GCN5: general control non-depressible 5
GSK3β: glycogen synthase kinase 3β
H2AX: H2A histone family member X
HATs: acetyltransferases
HDACs: deacetylases
HDR: homology-directed repair
HNF4: hepatocyte nuclear factor 4
HR: homologous recombination
Hsp70: heat shock protein 70
IDH2: isocitrate dehydrogenase 2
IFN-13/17: interleukin-13/17
IL-6: interleukin-6
IRS2: insulin receptor substrate 2
ISGF3: IFN-stimulated gene factor 3
JNK: c-Jun N-terminal kinase
KATs: lysine acetyltransferases
KDACs: lysine deacetylases
LARP7: La-related protein 7
LCAD: long-chain acyl-CoA dehydrogenase
LDHA: lactate dehydrogenase A
LKB1: live kinase B1
MAPK: mitogen-activated protein kinases
MDM2: murine double minute 2
MEF2: myocyte enhancer factor 2
MKP1: mitogen-activated protein kinase phosphatase 1
MMPs: matrix metalloproteinases
MnSOD: superoxide dismutase
mCAT: catalase targeted to the mitochondria
mTOR: mechanistic target of rapamycin
NHEJ: non-homologous end-joining
Nrf2: nuclear factor erythroid 2-related factor 2
PARP-1: poly (ADP-ribose) polymerase 1
PCAF: P300/CBP-associated factor
PD: Parkinson’s disease
PDK1: 3-phosphoinositide-dependent kinase 1
PEPCK1: Phosphoenolpyruvate carboxy-kinase
PGC-1α: peroxisome proliferator-activated receptor-gamma co-activator-alpha
PIK3C3: phosphatidylinositol 3-kinase catalytic subunit type 3
PIP3: phosphatidylinositol (3,4,5)-triphosphate
PKcs: DNA-dependent protein kinase catalytic subunit
PS1: presenilin-1
PT1B: protein tyrosine phosphatase 1B
PTEN: phosphatase and tensin homolog
PTMs: post-transcriptional modifications
PYCR1: pyrroline-5-carboxylate reductase 1
PYGO2: pygopus2
RAF: rapidly accelerated fibrosarcoma kinase
ROS: reactive oxygen species
RUBCN: RUN domain and Cysteine-rich domain containingBeclin 1-interacting protein
SAGA: Spt-Ada-Gen5 acetyltransferase
SASP: senescence-associated secretory phenotype
SIRT3: Sirtuins deacetylase family 3
SKP2: S-phase kinase-associated protein 2
Smad7: small mothers against decapentaplegic 7
SMC3: Structural maintenance of chromosomes 3
Smurf1: Sad ubiquitination regulatory factor 1
SOD2: superoxide dismutase 2
STAT3: signal transducer and activator of transcription 3
TFEB: transcription factor EB
TGF-β: transforming growth factor-beta
TIP60: Tat-interacting protein 60
TNF-α: tumor necrosis factor-alpha
TRIMs: tripartite motif proteins
UHRF1: ubiquitin-like with PHD and RING Finger domains 1
ULK1: UNC-52-like kinase 1
VSMCs: vascular smooth muscle cells
VPS34: vacuolar protein sorting 34
53BP1: tp53-binding protein 1
